# Investigation of the Effects of the COVID-19 Pandemic on Audiology Students in Turkey: A Cross-Sectional Survey Study in the COVID-19 Era

**DOI:** 10.3389/fpubh.2021.650981

**Published:** 2021-08-13

**Authors:** Gül Ölçek, İlayda Çelik, Yuşa Başoglu, Mustafa Bülent Serbetçioglu

**Affiliations:** Department of Audiology, Faculty of Health Sciences, Istanbul Medipol University, Istanbul, Turkey

**Keywords:** online education, Audiology students, COVID-19, e-learning, public health

## Introduction

In early 2020, after December 2019 outbreak in China, the World Health Organization (WHO) identified SARS-CoV-2 as a new type of coronavirus (COVID-19) (1). The COVID-19 announced as pandemic that occur severe acute respiratory syndrome and has not been previously identified in humans (1). Researches show that person-to-person transmission can occur via direct contact or through droplets spread by coughing or sneezing from an infected individual. All countries of the world have taken precautions against this virus, which has no effective treatment and spreads rapidly (2).

The first confirmed case in Turkey COVID-19 was recorded and declared on March 11, 2020. WHO announced COVID-19 outbreak as a pandemic on 11 March 2020 and the Turkish government took action with these developments thus restrictions, new implementations began in Turkey too (3). Restrictions such as distance learning or lockouts began to keep people at home longer and limit movement of the population.

The COVID-19 pandemic has affected the field of education as well as in other areas. In Turkey, as in many countries of the world, options such as various e-learning platforms that enable teachers and students to work and interact together, rapidly developing national television programs (e.g., TRT EBA TV, where primary, secondary and high school lessons are taught from distance) or lecture videos on social media platforms have started to be implemented (4).

COVID-19 pandemic rules and restrictions may have led to the prevalence of anxiety and depressive symptoms in the general population. Moreover, multiple stressors may have led to increased levels of stress, anxiety, and depressive thoughts among students. A study investigating the Mental Health of College Students showed that the COVID-19 pandemic had negative effects on higher education due to the prolonged pandemic situation and difficult measures such as lockdown and stay-at home orders (5). Nervousness, frustration, emotional confusion, sadness, exhaustion, boredom, insomnia, inadequate information, poor concentration, and indecisiveness, deteriorating work performance, financial problems are the most common psychological and behavioral reactions in this process (6).

Students' attitudes toward distance education may be variable. Too many factors may lead to these different attitudes. According to one study, although students express positive opinions about distance education, such as being comfortable with computer and internet use, feeling moderately effective and productive, and feeling moderate self-efficacy, they want to return to traditional education (7). This shows us that students struggle to fully adopt distance education.

Tele-health applications are also seen as a solution in Audiology to reduce the transmission of COVID-19. Audiologists are professionals who provide face-to-face patient care. During this period, one way of providing audiological services to patients was the transition to tele- Audiology service. The study of audiologists' opinions on tele-Audiology services during the COVID-19 pandemic showed that Audiologists generally had a positive experience (8). However, audiologists said that improvements and training in the system were needed and they also noted that some hybrid-care pathways should be available as some procedures need to be implemented in person (8). Tele-Audiology applications may offer some solutions for limited-service delivery capacity, especially during the COVID-19 pandemic (9). The use of different tele-Audiology service delivery models [synchronous real-time, asynchronous (store-and-forward), and hybrid models] can increase the accessibility of services (10). For these reasons, it is important to train Audiology students on tele-health and tele-Audiology issues during their education.

There has been a total and compulsory transition to the new system, because of the needs arising in terms of the education system during the COVID-19 period. E-learning (distance online education) was applied in this process in Turkey as a new model. However, this process brought with it difficulties in many areas such as education quality, e-learning, psychological and emotional state. In the literature, the effects of COVID-19 on education have been investigated, especially for applied departments. A study on the “Advantages and Limitations” of online learning during the COVID-19 pandemic was conducted with 12 students and 12 faculty members from the departments of medicine and dentistry. The study showed that while comfort and accessibility are among the advantages of online learning, limitations involved inefficiency and difficulty in providing academic integrity education (11). The study also stated that teaching and learning practical and clinical work through online learning modalities as the limit of online education (11). In a survey that ASHA conducted, 100% of audiology student participants and 98% of graduate speech-language pathology students showed that the pandemic had a “major” or “moderate” impact on their academic lives. E-learning can lead to difficulty in maintaining attention and ensuring interactive participation during the course (12). For this reason, we aimed to investigate this situation in many contexts. Furthermore, we planned to address not only the new system in education and the disadvantages it brought, but also the emotional psychological and social situations created by this period for students. Therefore, we determined the following questions in order to analyze the experiences of the Audiology students during the COVID-19 period: (1) readiness of universities to the new system, (2) course content, quality and level of interaction, (3) management of practical courses, (4) emotional, psychological, social and professional development status of students.

Audiology undergraduate program lasts 4 years, Master's program lasts 2 years, in Turkey. According to the National Audiology Main Education Program (for undergraduate program) audiology undergraduate education, practical courses start with the spring semester of the first year (13). There are also practical courses in the graduate education course period. The current study was conducted in the spring term of 2020.

The aim of the study is to examine the perspectives of Audiology students on online education, the level of competence of professional skills acquired by e-learning systems, and their psychological, emotional and social status during the COVID-19 pandemic in Turkey.

## Methods

**Figure 1:**
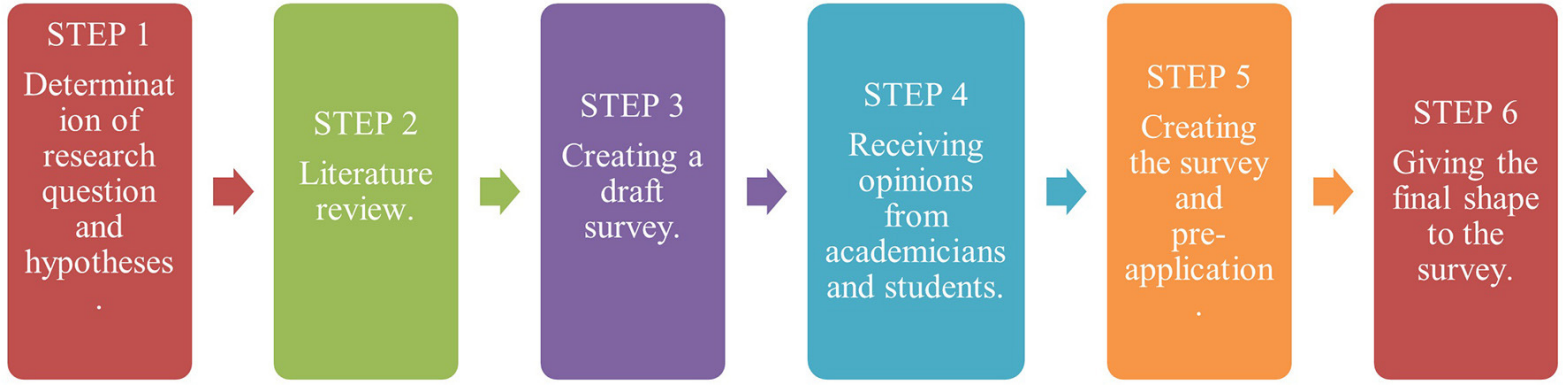
The research plan and the development process of the survey.

The study was performed between August 28 and September 30, 2020, in Turkey. The research was carried out using an online questionnaire created on Google Forms. Data were collected according to the 2020 spring semester when restrictions were most intense. First of all, survey questions were prepared taking into account the questions and opinions of each audiology class representative (students) and four academicians from the audiology department about the pandemic period. Then, we completed the survey arrangements, considering the guidelines of COVID-19 related associations such as American Speech-Language-Hearing Association (ASHA) (Figure 1) (14). The survey included demographic questions (4), common questions (25) and special questions for 4th grade students who new graduates are now (4). The questionnaire consists of three parts, the first part aimed to collect demographic information, while the second part aimed to collect data on whether the graduate students reached their career goals, whether they could find the job opportunities they wanted (4th grade in the 2020 spring semester), and the third part (common section) aimed to collect information about effect of COVID-19 pandemic on knowledge acquisition, social interaction, emotional state and academic performance competence. Detailed explanations about the purpose of the study, researchers and also the voluntary informed consent form were placed on the first page. While inviting the participants to the survey, a statement on the start page of the survey, “Our survey aims to examine the effects of the COVID-19 period with questions in different categories by shedding light on the problems experienced by audiology undergraduate and graduate students” emphasized that the participants should be audiology students.

Participation in the survey was completely voluntary, the informed consent form was marked from all participants thus all the participants willingly and voluntarily participated in the survey. After marking the consent form and declaring the acceptance of participation, the main questions were seen. A seven-point range scale was used to allow the participants to indicate how much they feel competency level with a specific statement and how much affected their specific situations during this period. So, two numerical scales with seven points were used in the dataset file. The first scale, where one is “too insufficient” and seven is “very sufficient” refers to participants' level of competence (1: too insufficient 2: insufficient 3: somewhat insufficient 4: neutral 5: somewhat sufficient 6: sufficient 7: very sufficient). The second scale, where 1 is “not at all” and 7 is “too much,” refers to the impact amount of the pandemic period on participants (1: not at all, 2: too little, 3: little, 4: neither less nor more, 5: a bit much, 6: much, 7: too much). (The questionnaire can be accessed online via the following links:

Turkish language: https://forms.gle/7qJQCNzBbwM7vKsTA,
English language: https://forms.gle/9Uz1yFzURu9asN948)

The main research questions of the current study were: (1) What is the educational satisfaction and content of the new (online education) system, in the COVID-19 era compared to the traditional (face-to-face education) system? (2) What competence do Audiology students see in themselves regarding practicing Tele-Audiology services, which is essential for the COVID-19 era. (3) What is the emotional, psychological and social impact of the COVID-19 conditions on Audiology students? (4) What are the effects of the distance education system on the personal and professional development of Audiology students? (5) Does sex, class degree and university type make any difference in terms of educational content, educational satisfaction, Tele-Audiology competence, psychological-social status and personal development status? (6) What are the conditions and competencies of newly graduated audiologists who graduate with distance education system in COVID-19 period?

We reached out to the participants through social media accounts (Instagram, Whatsapp etc.) and virtual office accounts (zoom, Microsoft teams etc.) that Audiology students subscribed to or followed. The survey was completed in approximately 10 min. The criterion for inclusion in the study was to be an Audiology undergraduate or graduate student. Descriptive statistics were used as statistical analysis with IBM SPSS 22.0 program. Descriptive data were calculated including mean values (M), median, interquartile range (IQR), standard deviations (SD), minimum and maximum values.

## Dataset

518 Audiology undergraduate and graduate students who studied Audiology at universities in Turkey participated in our survey in the spring semester of 2020. The 2020 spring semester was the period when undergraduate students had practical courses, and 2nd, 3rd, and 4th grades had practical courses and internships. Graduate students who were in the course period and clinical practice period in the 2020 spring semester were also included in the study. The respondents of this survey were from public and private universities in Turkey, and Table 1 shows the descriptive statistics of the demographic characteristics of these students. Demographic information consists of sex, class degree, university type (private / public) and reside area.

**Table 1:** Demographic information.

**Demographic information**	**The number of participants (%)**
**Sex**	
Female	452 (86.9)
Male	66 (13.1)
**University**	
Public University	150 (28)
Private University	368 (71)
**Reside area**	
Province	305 (58.9)
District	179 (34.6)
Rural	34 (6.6)
**Class degree**	
First grade student	145(28)
Sophomore	150 (29)
Third grade student	142(27.4)
Fourth grade student	67 (12.9)
Graduate student	14 (2.7)

Table 2, shows the outstanding results of the e-learning perspective, professional competence and tele-Audiology service perspective of the Audiology students during this period. There are findings about online education systems, distance education qualification and tele-Audiology service knowledge of Audiology students in the first section (from Q1 to Q19); also there are findings about psychological, social, emotional state and career goals of Audiology students during the COVID-19 pandemic period in the second section (from Q20 to Q25). In the last section (from Q26 to Q29), there are questions for 4th grade students. In this section, we asked how much new graduated Audiologists who graduated with distance education systems, due to the quarantine brought by the Covid-19 pandemic, achieved their career goals and the desired job. In addition, it was questioned whether new graduates from the Department of Audiology within the healthcare professional group have sufficient knowledge of COVID-19 rules (wearing masks, complying with social distance, hygiene rules) and sufficient professional knowledge to work in the field.

**Table 2:** Online survey descriptive statistics.

**Item**	**Questions (about online education system, distance education qualification and tele-Audiology service knowledge of Audiology students during the Covid-19 pandemic period)**	**N**	**Min (1: too insufficient)**	**Max (7: very sufficient)**	**Mean**	**SD**	**Median**	**IQR**
**Q1**	Please select the level of competence of technical equipment required for online education.	518	1	7	5.10	1.62	5.00	2.25
**Q2**	Please select your comfort level in using the online education platform.	518	1	7	4.79	1.65	5.00	2.00
**Q3**	Please select the level of your adaptation time to online education.	518	1	7	3.95	1.71	4.00	2.00
**Q4**	Please select the level of your ability to sustain your interest/attention to the lessons with online education.	518	1	7	3.39	1.73	3.00	3.00
**Q5**	Please select your level of interactive participation in online education.	518	1	7	3.72	1.82	4.00	3.00
**Q6**	Please select the level of professional knowledge you have acquired through the online theoretical courses.	518	1	7	3.47	1.73	3.00	3.00
**Q7**	Please select the level of professional knowledge you have acquired through the online practical courses.	518	1	7	2.98	1.77	3.00	3.00
**Q8**	Please select the adequacy of the exams in the form of homework in terms of the education system.	518	1	7	3.90	1.93	4.00	3.00
**Q9**	Please select the level of getting enough answers from academicians to your questions.	518	1	7	4.88	1.63	5.00	2.00
**Q10**	Please select the level of speed of the response time to your questions from academicians.	518	1	7	4.85	1.55	5.00	2.00
**Q11**	Please select your level of being able to devote yourself to online education exams or homeworks (your competence to work efficiently).	518	1	7	3.88	1.83	4.00	3.00
**Q12**	Please select the level of proficiency of your time spent on online education.	518	1	7	4.08	1.63	4.00	2.00
**Q13**	Please select the level of proficiency of the preparation duration for homework and exams.	518	1	7	4.00	1.76	4.00	2.00
**Q14**	Please select your level of readiness to internship/ work as Audiologist before the practical courses are completed.	518	1	7	2.54	1.63	2.00	3.00
**Q15**	Please select your level of professional development during the pandemic period.	518	1	7	3.11	1.65	3.00	2.00
**Q16**	Please select the contribution level of online seminars held during the pandemic period to your professional development.	518	1	7	3.72	1.75	4.00	3.00
**Q17**	Please select your level of knowledge about the precautions that should be applied in the clinic during a pandemic period.	518	1	7	3.32	1.72	3.00	3.00
**Q18**	Please select your level of theoretical knowledge about tele-Audiology services.	518	1	7	2.94	1.69	3.00	3.00
**Q19**	Please select your level of practical competence regarding tele-Audiology services.	518	1	7	2.64	1.65	2.00	3.00
**Item**	**Questions** (about psychological, social, emotional state and career goals of Audiology students during the Covid-19 pandemic period)	**N**	**Minimum (1: not at all)**	**Maximum (7: very much)**	**Mean**	**Std. Deviation**	**Median**	**Interquartile Range**
**Q20**	Please select your level of anxiety that occurred with the pandemic period.	518	1	7	0.53	1.51	6.00	2.00
**Q21**	Please select the level of psychological, social and emotional damage (that will require help) caused by this period.	518	1	7	4.02	0.87	4.00	3,00
**Q22**	Please select to what extent the pandemic process has negatively affected your social relationships.	518	1	7	5.09	1.74	5.00	3.00
**Q23**	Please select how well you spend your free time at home for your personal-social development during the pandemic period	518	1	7	3.91	1.64	4.00	2.00
**Q24**	Please select to what extent the pandemic period negatively affected your professional orientation and career goals.	518	1	7	4.68	1.72	5.00	2.00
**Q25**	Please select your level of satisfaction with the distance education period you experience with the pandemic process, taking all factors into account	518	1	7	3.52	1.70	4.00	3.00
**Q26**	Please select the level of achieving your career goals in this process.	67	1	7	4.07	1.63	4.00	2.00
**Q27**	Please select the level of competence of your knowledge about what precautions you need to take in your workplace (wearing masks, gloves, social isolation, etc.) when you start working.	67	4	7	6.28	0.83	6.00	1.00
**Q28**	Please select the level of competence of your professional skills that you feel yourself sufficient to work as an Audiologist in the pandemic period.	67	1	6	4.19	1.23	4.00	2.00
**Q29**	Please select the level of starting the job you want as a result of the job opportunities affected by this process.	67	1	7	3.57	1.58	4.00	3.00

*The reliability of the survey was evaluated by Cronbach's alpha, where the sufficiency level for the alpha coefficient is ≥0.70 (Cortina 1993). In this research, Cronbach's alpha coefficient for the whole survey was 0.83, which shows good internal consistency. Moreover, Cronbach's alpha value for ‘’E-learning, Tele-Audiology, psychological-emotional-social status and personal development” subscales was found to be 0.93, 0.89, 0.81, and 0.73, respectively*.

## Strengths and Limitations

The current dataset has several strengths. Important data was obtained from 518 students during the survey access process, which took about a month. The dataset is important as the number of participants represents ~22% of all audiology students (2,355) in 2019-2020 (15). However, the participation of a small number of graduate students can be seen as a limitation of the study. Another strength of this dataset is that it is the first survey to show the effects of the pandemic on Audiology students from Turkey.

Furthermore, current dataset shows the multiple effects of COVID-19 on Audiology students, such as the level of professional knowledge acquisition of the Audiology students through online education and their personal-social-emotional state during this period.

Another limitation of this dataset may be that we use our own survey, which we have created according to the COVID-19 period, rather than a specific scale accepted in the literature.

The data are useful for other researchers and educators to see the effects of this period on students studying in health departments that include applied clinical courses such as Audiology. Data set comparison is important in terms of comparing other data sets collected from other countries with other datasets that examine the effect of both online and face-to-face education on students' educational status.

The dataset can serve as a reference source for seeing students' situations during distance education for health departments which includes applied clinical courses. Moreover, this study can provide valuable information about the courses and students' attitudes toward distance health services such as tele-Audiology.

In the light of the information obtained from this dataset, improvement strategies can be created in the education system for applied departments such as Audiology.

## Possible Research Paths

The dataset provides information about Audiology students' perspective on distance online education in the spring semester of the 2019-2020 academic year (17 March-29 May 2020), the effects of e-learning systems on professional skill acquisition, and emotional, psychosocial status of the students during the pandemic. The dataset may be divided by sex, class degree, university type and area of residence. Moreover, the dataset may be used to compare with these different demographics. For example, the third academic year, a year in which the content of education is intense, may have been more difficult for students during this period.

In future research, this data set could also shed light on universities with Audiology departments in other countries. Also, the dataset can be used to provide recommendations during the current pandemic or to provide preliminary insight into possible quarantine restrictions in future pandemics.

## Dataset Description

The survey form was submitted through an online platform. The link to the online survey was distributed to students using several social media and e-learning platforms, such as WhatsApp groups, Instagram, Microsoft Teams.

## Data Availability Statement

The datasets for this study can be found at https://doi.org/10.17632/j36zbmf4b4.1.

## Ethics Statement

The studies involving human participants were reviewed and approved by Ethics committee approval was obtained from the Istanbul Medipol University Non-Interventional Clinical Studies Ethics Committee with reference number 10840098-772.02-E.36005, dated 10.08.2020. Participation in the survey was completely voluntary, the online informed consent form was marked from all participants thus all the participants willingly and voluntarily participated in the survey. The patients/participants provided their written informed consent to participate in this study.

## Author Contributions

GÖ designed the study, collected the data, and wrote and edited the text. İÇ designed the study, collected the data, contributed to the analysis, and wrote the text. YB designed the analysis, performed the analysis, and wrote the text. MS edited the text and wrote the text. All authors listed approved the study for publication.

## Conflict of Interest

The authors declare that the research was conducted in the absence of any commercial or financial relationships that could be construed as a potential conflict of interest.

## Publisher's Note

All claims expressed in this article are solely those of the authors and do not necessarily represent those of their affiliated organizations, or those of the publisher, the editors and the reviewers. Any product that may be evaluated in this article, or claim that may be made by its manufacturer, is not guaranteed or endorsed by the publisher.
